# Identification of Phonology-Related Genes and Functional Characterization of Broca’s and Wernicke’s Regions in Language and Learning Disorders

**DOI:** 10.3389/fnins.2021.680762

**Published:** 2021-09-03

**Authors:** Nina Unger, Stefan Heim, Dominique I. Hilger, Sebastian Bludau, Peter Pieperhoff, Sven Cichon, Katrin Amunts, Thomas W. Mühleisen

**Affiliations:** ^1^Cécile and Oskar Vogt Institute for Brain Research, Medical Faculty, University Hospital Düsseldorf, Heinrich Heine University Düsseldorf, Düsseldorf, Germany; ^2^Institute of Neuroscience and Medicine (INM-1), Research Centre Jülich, Jülich, Germany; ^3^Department of Neurology, Medical Faculty, RWTH Aachen University, Aachen, Germany; ^4^Department of Psychiatry, Psychotherapy and Psychosomatics, Medical Faculty, RWTH Aachen University, Aachen, Germany; ^5^JARA-Brain, Jülich-Aachen Research Alliance, Jülich, Germany; ^6^Department of Biomedicine, University of Basel, Basel, Switzerland; ^7^Institute of Medical Genetics and Pathology, University Hospital Basel, Basel, Switzerland

**Keywords:** development, phonology, gene expression, Broca’s area, Wernicke’s area

## Abstract

Impaired phonological processing is a leading symptom of multifactorial language and learning disorders suggesting a common biological basis. Here we evaluated studies of dyslexia, dyscalculia, specific language impairment (SLI), and the logopenic variant of primary progressive aphasia (lvPPA) seeking for shared risk genes in Broca’s and Wernicke’s regions, being key for phonological processing within the complex language network. The identified “phonology-related genes” from literature were functionally characterized using Atlas-based expression mapping (JuGEx) and gene set enrichment. Out of 643 publications from the last decade until now, we extracted 21 candidate genes of which 13 overlapped with dyslexia and SLI, six with dyslexia and dyscalculia, and two with dyslexia, dyscalculia, and SLI. No overlap was observed between the childhood disorders and the late-onset lvPPA often showing symptoms of learning disorders earlier in life. Multiple genes were enriched in Gene Ontology terms of the topics learning (*CNTNAP2, CYFIP1, DCDC2, DNAAF4, FOXP2*) and neuronal development (*CCDC136, CNTNAP2, CYFIP1*, *DCDC2*, *KIAA0319*, *RBFOX2*, *ROBO1*). Twelve genes showed above-average expression across both regions indicating moderate-to-high gene activity in the investigated cortical part of the language network. Of these, three genes were differentially expressed suggesting potential regional specializations: *ATP2C2* was upregulated in Broca’s region, while *DNAAF4* and *FOXP2* were upregulated in Wernicke’s region. *ATP2C2* encodes a magnesium-dependent calcium transporter which fits with reports about disturbed calcium and magnesium levels for dyslexia and other communication disorders. *DNAAF4* (formerly known as *DYX1C1*) is involved in neuronal migration supporting the hypothesis of disturbed migration in dyslexia. *FOXP2* is a transcription factor that regulates a number of genes involved in development of speech and language. Overall, our interdisciplinary and multi-tiered approach provided evidence that genetic and transcriptional variation of *ATP2C2*, *DNAAF4*, and *FOXP2* may play a role in physiological and pathological aspects of phonological processing.

## Introduction

Developmental language and learning disorders severely impair children’s abilities in speaking, reading, writing, calculating, and combinations thereof (American Psychiatric Association, 2013). One cognitive domain commonly involved in the majority of these disorders is phonological processing which refers to the analysis and synthesis of the sound structure of spoken and written language (phonological awareness), phonological representations and the rapid access (rapid automatized naming) or memorizing (phonological working memory) thereof. The different phonological domains are highly inter-correlated and part of the shared construct of phonological processing.

At the behavioral level, patients with dyslexia, dyscalculia, or specific language impairment (SLI) show deficits in key domains of phonological processing such as phonological awareness, phonological working memory, and/or speeded access to phonological codes during rapid automatized naming, as summarized in Supplementary Table 1. Dyslexia is primarily associated with impaired phonological awareness (Steinbrink et al., 2008; Pennington and Bishop, 2009; Hasselhorn et al., 2010; Child et al., 2019). This, like rapid automatized naming, may be considered as a predictive factor for early-onset dyslexia (Thompson et al., 2015). In addition, dyslexia patients perform poorly on phonological working memory (De Carvalho et al., 2014; Child et al., 2019) and show deficits in phonological representations leading to difficulties in phoneme awareness and phonological coding (Pennington and Bishop, 2009). In dyscalculia, connections between phonological processing and arithmetic abilities can be observed (De Smedt and Boets, 2010; Raghubar et al., 2010; De Smedt, 2018). The phonological working memory was identified to serve as a short-time buffer for mathematical operations based on the triple code model which codes numbers as numerals, number words, and abstract numerosities (Dehaene, 1992; Dehaene et al., 2003; Träff and Passolunghi, 2015; Pollack and Ashby, 2018). Patients with SLI, especially with a speech sound disorder, exhibit an inadequate phonological realization and use of particular phonemes in spontaneous speech due to impaired phonological awareness abilities and a lower quality of phonological representations (Lewis et al., 2011; Claessen and Leitão, 2012; American Psychiatric Association, 2013). Some studies also showed lower scores in SLI in phonological working memory increasing the language difficulties (Torrens and Yagüe, 2016).

In addition to impaired phonological processing with effects on language, reading, and mathematical abilities early in life, phonological deficits may have an onset late in life, e.g., in patients with primary progressive aphasia, a progressive neurodegenerative disease (Magnin et al., 2016). The logopenic variant of primary progressive aphasia (lvPPA) was included in this research for this reason and due to high comorbidity with developmental learning disorders meaning that lvPPA patients have a higher probability for these disorders in childhood (Rogalski et al., 2008). Like children with SLI, adults with lvPPA exhibit phonological paraphasias, i.e., errors in the phonological realization of a word, in spontaneous speech as well as in naming (Gorno-Tempini et al., 2011). One central mechanism for these lvPPA symptoms is a deficit of phonological working memory. In addition, alterations in phonological representations may be observable (Gorno-Tempini et al., 2008; Henry et al., 2016).

Phonological processing is related to the language areas of the fronto-temporal network, in particular Broca’s region (opercular and triangular parts of the left inferior frontal gyrus) and Wernicke’s region (left inferior parietal lobule and posterior superior temporal gyrus; Müller and Knight, 2006; Rogalsky and Hickok, 2011; Hartwigsen, 2015, 2016; Binder, 2017; Klaus and Hartwigsen, 2019; Yi et al., 2019; Hartwigsen et al., 2020). Broca’s and Wernicke’s regions represent central nodes of the phonological processing network, which are integrated into phonological processes in phonological production as well as in phonological perception (Heim et al., 2003; Indefrey and Levelt, 2004; Friederici, 2011; Indefrey, 2011; Hagoort and Indefrey, 2014). While Broca’s region is associated with phonological word fluency, phonological decisions, and the phonological loop (Heim et al., 2008, 2009a,b; Aboitiz et al., 2010; Liakakis et al., 2011; Katzev et al., 2013; Wagner et al., 2014; Klaus and Hartwigsen, 2019), Wernicke’s region is involved in phonological speech perception and auditory word-form recognition (Buchsbaum et al., 2001; DeWitt and Rauschecker, 2013). Both regions are connected by the language-relevant pathways, arcuate fasciculus and superior longitudinal fasciculus (Catani et al., 2005; Friederici, 2009, 2011, 2012; Friederici and Gierhan, 2013). Both fasciculi are associated with the transport of order information as a part of the phonological loop (Papagno et al., 2017) and the arcuate fasciculus is also involved in automatic repetition (Catani et al., 2005; Berthier et al., 2012; Papagno et al., 2017). Furthermore, a high functional connectivity between Broca’s and Wernicke’s regions based on the arcuate fasciculus leads to a higher linguistic performance, e.g., in phonological word learning (López-Barroso et al., 2013).

Functional resonance imaging and diffusion tensor imaging studies suggested that Broca’s and Wernicke’s regions are affected in patients with SLI, dyslexia, dyscalculia, and also the lvPPA (McCrone, 2002; Sonty et al., 2007; Heim et al., 2010; Verhoeven et al., 2012; Dinkel et al., 2013). In children with SLI, an abnormal connection between both regions was reported in contrast to age-matched controls, i.e., a significantly reduced mean fractional anisotropy was shown in the superior longitudinal fasciculus (Verhoeven et al., 2012). Dyslexia patients showed lower activations in Broca’s region during phonological decision tasks as well as in Wernicke’s region during auditory discrimination tasks (Heim et al., 2010). In dyscalculia patients, both regions are the common regions for the interaction of phonological processing and mathematical abilities (Pollack and Ashby, 2018). Additionally, reduced connectivity between Wernicke’s region and the intraparietal sulcus was found in dyscalculia patients (McCrone, 2002). Moreover, Broca’s region was excessively activated in dyscalculia patients during a calculation task (Dinkel et al., 2013). In lvPPA patients, Sonty et al. (2007) reported a lower effective connectivity (dysfunctional network interaction) between Broca’s and Wernicke’s regions.

Twin studies suggest that reading and language skills are heritable, with influences from both genetic factors and environmental factors (Hensler et al., 2010; Wadsworth et al., 2010; Tosto et al., 2017; Rice et al., 2020). Similar to developmental disorders, primary progressive aphasia also has a genetic component (Henry and Gorno-Tempini, 2010; Kim et al., 2016). Yet, there are no systematic approaches published that relate genes to the domains of phonological processing, but rather only to isolated phonological features within language and/or learning disorders or the physiological language. Furthermore, there is no study about a specific expression analysis of language-related genes in Broca’s and Wernicke’s regions of the adult human brain. However, two studies investigated the developing brain using data-driven, whole-transcriptome approaches to identify profiles of regionally co- or differentially expressed genes. Johnson et al. (2009) screened different brain regions in human fetuses including the perisylvian cortex (ventrolateral prefrontal, motor-somatosensory, parietal association, temporal auditory), where *FOXP2* showed a modest upregulation. Lambert et al. (2011) directly screened precursor structures of Broca’s and Wernicke’s regions in the fetal brain and found several *FOXP2* regulated genes among the differentially expressed genes. An overview of the other recent studies on Broca’s and Wernicke’s regions in humans and model organisms is provided by Supplementary Table 2.

**FIGURE 1:**
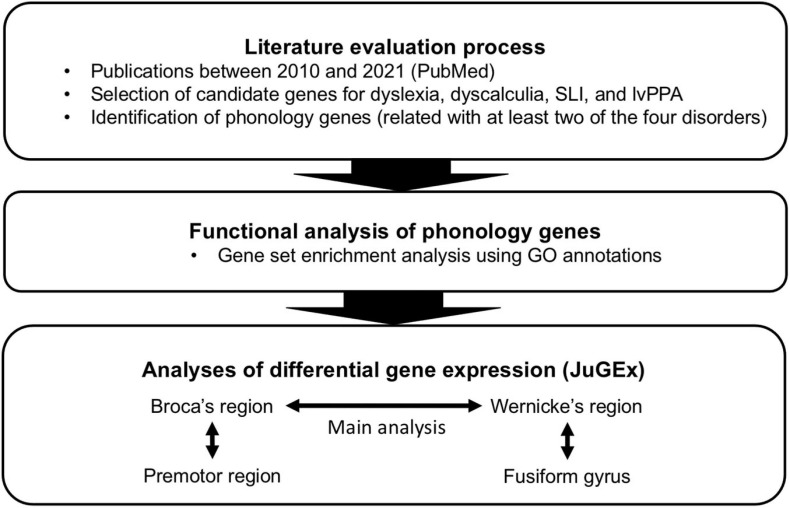
Design of the present study.

Given that the named language and/or learning disorders in childhood and late adulthood have commonalities in (a) phonological processing deficits as one cognitive signature, (b) repercussions in the neural processing circuits for phonology, and (c) genetic predispositions, we wanted to investigate whether there is a specific genetic contribution to general phonological processing principles for dyslexia, dyscalculia, SLI, and lvPPA. For this purpose, we developed a workflow to integrate clinical, neuroanatomical, and genetic knowledge about phonological processing abilities in these disorders (Figure 1). First, we performed a screening of the literature for potentially candidate genes for phonological processing (“phonology-related genes”) shared between the disorders. We then characterized these genes by enrichment analysis of gene functions and expression analysis in the two key regions involved in phonological processing (Broca’s and Wernicke’s regions) using a new computational tool (JuGEx, Bludau et al., 2018) for the joint statistical analysis of microstructural variability (Julich-Brain Atlas; Amunts et al., 2020) and transcriptional variability (Hawrylycz et al., 2012, 2015; Allen Human Brain Atlas, 2013) in adult human brains.

## Materials and Methods

### Literature Evaluation Process

The following criteria were used to screen the literature and had to be fulfilled: (a) Publication date between 01/01/2010 and 23/05/2021, (b) investigation of at least one of the disorders dyslexia, dyscalculia, SLI, and lvPPA, (c) report of at least one human gene as significant original/replicated result (*p* < 0.05), (d) report of at least one gene as a risk factor for a disorder or trait/phenotype related with the disorder, (e) sample containing more than one individual (no single-case study), (f) sample of European origin to reduce a potential influence of population stratification on our results, (g) study either a linkage analysis, a genome-wide association study (GWAS), or a candidate gene association study.

Criterion (b) was not met if a disorder was named as an attendant symptom of another disorder, e.g., dyslexia as an attendant symptom of tuberous sclerosis. In contrast, articles primarily dealing with specific language and/or learning disorders with additional comorbidities were included, e.g., dyslexia with comorbid attention-deficit hyperactivity disorder. Our criteria referred to the terminology defined by the two most recent Diagnostic and Statistical Manuals of the American Psychiatric Association [DSM-IV (American Psychiatric Association, 1994); DSM-5 (American Psychiatric Association, 2013)] and exact synonyms. Studies investigating the normal variability of a trait in a population sample, e.g., reading skills in a cohort of healthy children, were only included if a direct relation to a corresponding pathology was reported, e.g., in terms of the analysis of risk genes for that pathology.

We performed eleven single searches fully covering the potential combinations of language and/or learning disorders (Supplementary Table 3) using PubMed Advanced Search^1^ with our search string, composed of clinical categories and genetic terms as described in Supplementary Table 4. Each search consisted of two analytical steps: First, a computational text mining of the abstracts and titles from all identified articles using PubTator^2^ (Wei et al., 2013). Second, a manual text mining of the full text was performed. Articles were selected for inclusion if they fulfilled all criteria a) to g). Clinical categories found in these articles were summarized in Supplementary Table 5. Candidate genes were extracted from research articles, while review articles were excluded from evaluation results because they did not represent original research results. Symbols and names of genes were checked and edited, if necessary, in order to match definitions by the Human Genome Nomenclature Committee^3^. To identify phonology-related genes, we looked for genes that were reported for more than one of the disorders using a Venn diagram tool^4^ : A phonology-related gene had to be involved in at least two of the four disorders (overlaps).

### Functional Enrichment Analysis

Genes were annotated by gene ontology (GO; Gene Ontology Consortium, 2021) using ToppFun, a tool of the ToppGene Suite^5^ (Chen et al., 2009). ToppFun was used to find out which GO terms and identifiers (as of 12/03/2021) from the categories “Biological Process,” “Molecular Function,” or “Cellular Component” were over-represented (enriched) in the list of phonology-related genes from the Venn diagram intersections. The following analytical parameters were chosen: hypergeometric probability mass function as *p*-value method, Bonferroni corrected *q*-value < 0.05, gene limits 1 ≤ *n* ≤ 2000, 2 as minimum feature count in the test set. To interpret the relationships of the identified GO terms, we used ancestor charts from QuickGo^6^. These charts are conceptual figures to represent a tree of hierarchically linked GO terms (parental and child terms). Under the same parameters, an enrichment analysis with different pathway annotations was performed with ToppFun which did not yield biologically meaningful significant results possibly due to the small number of candidate genes and/or limited functional annotations for these genes.

### Gene Expression Analysis in Cytoarchitectonically Defined Brain Areas

Gene expression differences between pairs of brain regions, technically referred to as volumes of interest (VOIs), were analyzed using the workflow and graphical user interfaces (GUIs) of JuGEx (Bludau et al., 2018). The tool is based on scripts coded in MATLAB (version R2018b, 64 bit; The MathWorks) which are freely accessible (available online^7^). JuGEx integrates regional RNA data from the Allen Human Brain Atlas from three right-handed, two left-handed, and one ambidextrous donors (Hawrylycz et al., 2012) with the three-dimensional cytoarchitectonic probability maps from the Julich-Brain Atlas (Amunts et al., 2020) which are based on observer-independent mapping of cytoarchitectonic areas in ten postmortem brains. Allen Human Brain Atlas and Julich-Brain data are based on brain tissue from deceased adult donors without a psychiatric or neurological diagnosis; the brains did not overlap between both atlas systems (Bludau et al., 2018). The expression data derived from anisotropically distributed tissue samples of Allen Human Brain Atlas and cytoarchitectonic maps from Julich-Brain were registered to the reference space of the Montreal Neuroscience Institute 152 (MNI_152_) reference brain (Evans et al., 2012). To enable a comparison of gene expression between VOIs in different brains, JuGEx uses relative expression values that were normalized across all regions and six brains from Allen Human Brain Atlas using z-scores (Hawrylycz et al., 2015).

A gene was investigated using the “all-probes mode” which calculates a winsorized mean based on the microarray probes for each gene (Bludau et al., 2018). We considered a *p*-value smaller than 0.05 as a significant difference between the z-scores of tissue samples in the compared VOIs. The nominal *p*-values from the permuted n-way analysis of variance (ANOVA) were corrected for multiple comparisons by applying a family-wise error (FWE) correction with the number of analyzed genes (*n* = 21, *p*_FWE_ < 0.05). The permuted n-way ANOVA was repeated 10,000 times with randomly shuffled labels of the analyzed VOIs under the assumption of label exchangeability. The regional specificity of a gene was defined as significantly higher expression (z-score) in that area (first VOI) compared to another area (second VOI).

Main analysis: In order to define “Broca’s region” anatomically, the maps of areas 44 and 45, both cytoarchitectonically described by Amunts et al. (1999, 2004), were merged using JuGEx. We decided to merge both areas considering the spatial resolution of gene expression data in Allen Human Brain Atlas with rather large intervals between sections in relation to the size of the areas. The Te3 map of the Julich-Brain Atlas was used as a proxy for “Wernicke’s region,” a cytoarchitectonically defined area located in the lateral bulge of the superior temporal gyrus (Morosan et al., 2005). Te3 overlaps in parts with Brodmann’s area 22 as defined by Brodmann (1909). Since the left hemisphere is dominant for language in most right- and left-handers (Branch et al., 1964; Mazoyer et al., 2014), we investigated the activity (expression) of potentially functionally relevant candidate genes in the left hemispheres.

Regional specification analysis: To characterize the regional specificity in the frontal lobe, the Julich-Brain maps of premotor areas 6d1, 6d2, and 6d3 were combined and compared with Broca’s region. The same was done in the temporal lobe by merging the maps Fg1, Fg2, Fg3, and Fg4 of the fusiform gyrus and by comparing them with Wernicke’s region. We considered support for regional specificity if the candidate gene was not significantly upregulated in the non-language areas.

Control analysis: To further evaluate the main analysis, another comparison between Broca’s and Wernicke’s regions was performed with two additional gene sets. The first set was chosen from the Allen Human Brain Atlas genes and comprised 25 genes, hereafter called “random genes” (Supplementary Table 6A). The second set was intentionally taken from the Allen Human Brain Atlas genes and included 14 genes from GWAS of eye, hair, and skin coloration, hereafter called “color genes” (Supplementary Table 6B). Both random and color genes were assembled and considered as biological negative controls as described by Bludau et al. (2018).

The following numbers of tissue samples were identified through the aforementioned VOIs in the six brains from Allen Human Brain Atlas using a Julich-Brain map threshold of 20% (this means that only tissue samples were included with a probability of 20% or higher belonging to the corresponding VOIs): left areas 44 and 45 (*n*_tissue samples_ = 25), left area Te3 (*n*_tissue samples_ = 17), left areas 6d1, 6d2, and 6d3 (*n*_tissue samples_ = 30), left areas Fg1, Fg2, Fg3, and Fg4 (*n*_tissue samples_ = 57). Before statistical analyses, the spatial assignment of tissue samples to maps was manually checked using the “3D visualization” GUI of JuGEx.

The full data of the used maps are provided by the Julich-Brain Atlas *via* the Human Brain Project (available online^8^): Areas 44 (doi: 10.25493/N13Y-Y3F), 45 (doi: 10.25493/K06P-R2S), Te3 (doi: 10.25493/ZZQH-932), 6d1 (doi: 10.25493/KSY8-H3F), 6d2 (doi: 10.25493/WJQ5-HWC), 6d3 (doi: 10.25493/D41S-AG7), Fg1 (doi: 10.25493/RTDM-GK4), Fg2 (doi: 10.25493/N7ZP-17X), Fg3 (doi: 10.25493/F87E-JRA), and Fg4 (doi: 10.25493/MTWF-X4V).

## Results

### Phonology-Related Genes From Literature

**FIGURE 2:**
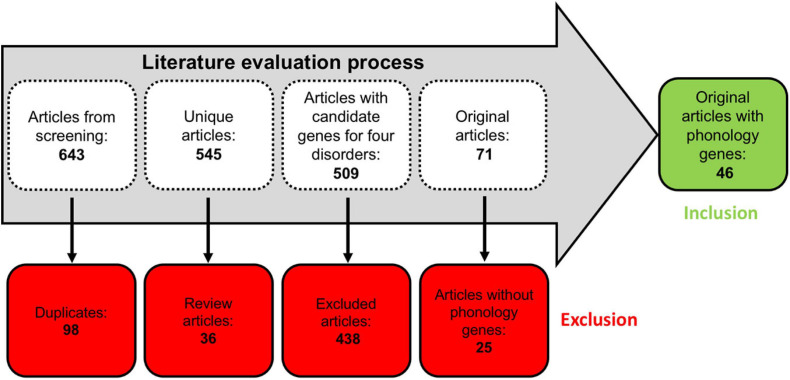
Workflow of the literature evaluation process.

**FIGURE 3:**
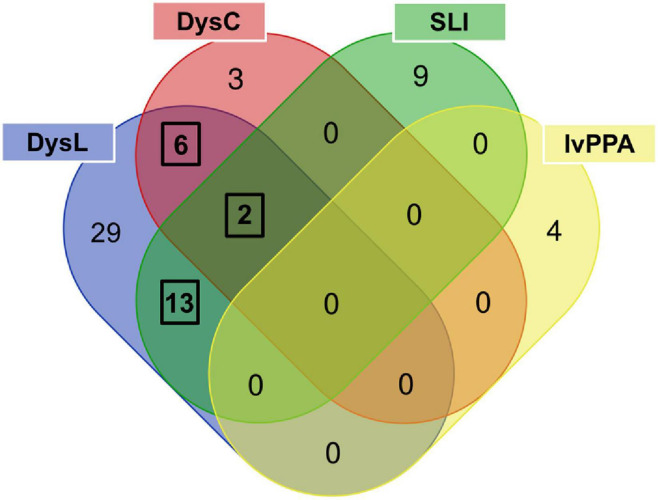
Venn diagram used to determine phonology-related genes from candidate genes overlapping between language and/or learning disorders. The three black frames indicate overlaps of genes associated with at least two disorders. DysL, dyslexia; DysC, dyscalculia; SLI, specific language impairment; lvPPA, logopenic variant of primary progressive aphasia.

The literature evaluation process comprised two main steps, a computational text mining of titles, abstracts, and key words, followed by a manual text mining of the whole text body and supplement, to find original research articles and genes (Figure 2). The computational text mining of dyslexia, dyscalculia, SLI, and lvPPA literature from the last 11 years using search strings of clinical categories and genetic terms (Supplementary Table 4) revealed 643 journal articles. Of these, 98 articles were found more than once, and additional 36 articles were excluded since they were reviews (Supplementary Table 7A). Thus, 509 unique research articles provided the basis for further manual text mining which led to the exclusion of another 438 articles that were filtered out with PubTator or did not meet the above mentioned inclusion criteria at whole-text screening. Eventually, 71 research articles remained reporting 77 potential candidate genes (Supplementary Tables 7B,C). For the final selection, we put the information of Supplementary Table 7C forward to a Venn diagram to seek for overlapping candidate genes from the different disorders (Figure 3).

For dyslexia/SLI, we found *ATP2C2* (Newbury et al., 2011; Müller et al., 2017; Martinelli et al., 2021), *CCDC136* (Gialluisi et al., 2014; Adams et al., 2017), *CMAHP* (Eicher et al., 2014), *CMIP* (Newbury et al., 2011; Scerri et al., 2011; Luciano et al., 2018), *CNTNAP2* (Newbury et al., 2011; Peter et al., 2011; Eicher et al., 2013; Luciano et al., 2018), *COL4A2* (Eicher et al., 2013), *FLNC* (Gialluisi et al., 2014; Adams et al., 2017), *FOXP2* (Peter et al., 2011; Wilcke et al., 2012; Eicher et al., 2013; Mozzi et al., 2017; Sánchez-Morán et al., 2018; Doust et al., 2020), *KIAA0319* (Couto et al., 2010; Czamara et al., 2011; Elbert et al., 2011; König et al., 2011; Newbury et al., 2011; Scerri et al., 2011; Cope et al., 2012; Zou et al., 2012; Eicher et al., 2013, 2014; Powers et al., 2013, 2016; Mascheretti et al., 2015; Adams et al., 2017; Carrion-Castillo et al., 2017; Neef et al., 2017; Centanni et al., 2018; Luciano et al., 2018; Sánchez-Morán et al., 2018), *NOP9* (Pettigrew et al., 2016), *RBFOX2* (Gialluisi et al., 2014), *RIPOR2* (Eicher et al., 2014), and *ZNF385D* (Eicher et al., 2013). For dyslexia/dyscalculia, we observed *CYFIP1* (Ulfarsson et al., 2017), *DNAAF4* (Bates et al., 2010; Marino et al., 2011; Paracchini et al., 2011; Mascheretti et al., 2013, 2015; Müller et al., 2017; Luciano et al., 2018), *MYO18B* (Ludwig et al., 2013), *NIPA1* (Ulfarsson et al., 2017), *NIPA2* (Ulfarsson et al., 2017), and *TUBGCP5* (Ulfarsson et al., 2017). For dyslexia/dyscalculia/SLI, we found *DCDC2* (Lind et al., 2010; Czamara et al., 2011; König et al., 2011; Marino et al., 2011, 2012, 2014; Newbury et al., 2011; Scerri et al., 2011; Cope et al., 2012; Eicher et al., 2013, 2014, 2015; Powers et al., 2013, 2016; Cicchini et al., 2015; Gori et al., 2015; Matsson et al., 2015; Adams et al., 2017; Neef et al., 2017; Luciano et al., 2018; Riva et al., 2019) and *ROBO1* (Bates et al., 2011; Mascheretti et al., 2014; Tran et al., 2014; Luciano et al., 2018). From the final selection of phonology-related candidate genes (Supplementary Table 7D), the most significant genetic markers of each gene reported by the original study were extracted and summarized in Supplementary Table 7E.

### Enrichment Analysis of Phonology-Related Genes

All candidate genes were analyzed for statistical over-representation in GO using ToppFun and the original ancestor charts from GO (Methods section “Functional Enrichment Analysis”). Eleven GO terms showed significant enrichments of phonology-related gene sets that withstood a correction for multiple comparisons (Table 1). Total results of this analysis including terms that did not withstand correction are described in Supplementary Table 8. The most significant result of the enrichment analysis was the “Biological Process” term “response to auditory stimulus” from the topic *learning*, where we found an enrichment of *CNTNAP2*, *FOXP2*, and *KIAA0319* out of 28 genes from this term (GO:0010996). In hierarchical relationships to this were the child terms “vocal learning” and “response to auditory behavior,” where the genes *CNTNAP2* and *FOXP2* were enriched. Within the top eleven results were three additional GO terms which support *learning*. They form a group of terms (cluster), where the neighboring cluster starts at the parent term “cognition” (GO:0050890; enriched genes *CNTNAP2, CYFIP1*, *DCDC2*, *DNAAF4, FOXP2*), went over “observational learning” (GO:0098597) and “imitative learning” (GO:0098596) and finally ended this GO term path in “vocal learning.”

**TABLE 1:** Results of the functional enrichment analysis of phonology-related genes.

Term name	Term identifier	GO Category	Hit count query/genome	Phonology-related genes in term genes	*p*-value^#^
Response to auditory stimulus	GO:0010996	Biological Process	3/28	*CNTNAP2, FOXP2, KIAA0319*	**2.67E-06**
Dendrite development	GO:0016358	Biological Process	5/310	*CYFIP1, DCDC2, KIAA0319, RBFOX2, ROBO1*	**1.05E-05**
Cellular component morphogenesis	GO:0032989	Biological Process	7/914	*CCDC136, CNTNAP2, CYFIP1, DCDC2, KIAA0319, RBFOX2, ROBO1*	**1.75E-05**
Vocal learning	GO:0042297	Biological Process	2/7	*CNTNAP2, FOXP2*	**1.95E-05**
Imitative learning	GO:0098596	Biological Process	2/7	*CNTNAP2, FOXP2*	**1.95E-05**
Cognition	GO:0050890	Biological Process	5/375	*CNTNAP2, CYFIP1, DCDC2, DNAAF4, FOXP2*	**2.64E-05**
Observational learning	GO:0098597	Biological Process	2/9	*CNTNAP2, FOXP2*	**3.34E-05**
Cell morphogenesis involved in neuron differentiation	GO:0048667	Biological Process	6/715	*CYFIP1, DCDC2, KIAA0319, RBFOX2, RIPOR2, ROBO1*	**4.86E-05**
Neuron projection membrane	GO:0032589	Cellular Component	3/73	*CNTNAP2, RIPOR2, ROBO1*	**5.69E-05**
Magnesium ion transmembrane transporter activity	GO:0015095	Molecular Function	2/16	*NIPA1, NIPA2*	**1.33E-04**
Axolemma	GO:0030673	Cellular Component	2/21	*CNTNAP2, ROBO1*	**2.12E-04**

*^#^*P*-value of the underlying hypergeometric probability mass function.*

*Bold: *p*-value stable against correction for multiple comparisons (Bonferroni adjusted *q*-value < 0.05).*

The second and third most significant GO terms were “dendrite development” (GO:0016358), enriched by *CYFIP1*, *DCDC2*, *KIAA0319*, *RBFOX2*, and *ROBO1*, and “cellular component morphogenesis” (GO:0032989), enriched by *CCDC136, CNTNAP2, CYFIP1, DCDC2, KIAA0319, RBFOX2*, and *ROBO1*, both pointing to *neuronal development* as the second big topic of the enrichment analysis. This finding was complemented by the two terms from the GO category “Cellular Component” “neuron projection membrane” (GO:0032589) and “axolemma” (GO:0030673) and the “Biological Process” category term “cell morphogenesis involved in neuron differentiation” (GO:0048667). The only GO term from the “Molecular Function” category among our significant results was “magnesium ion transmembrane transporter activity” (GO:0015095).

### Expression of Phonology-Related Genes in Broca’s and Wernicke’s Regions

**FIGURE 4:**
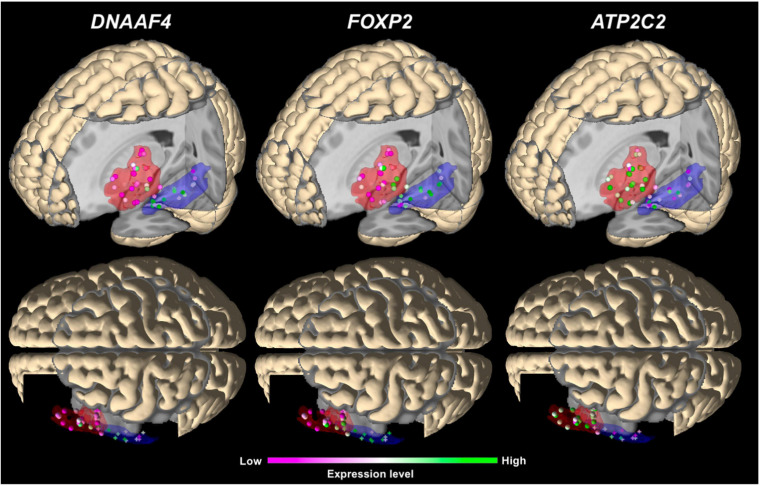
3D visualization of the main expression analysis using JuGEx in lateral views (top panels) and dorsal views (bottom panels) displaying the three significantly differentially expressed genes: *DNAAF4* and *FOXP2* show higher expression (FWE-corrected) in Wernicke’s region and *ATP2C2* in Broca’s region. Investigated tissue samples are represented by spheres in the red areas (Broca’s region) and crosses in the blue areas (Wernicke’s region). RNA expression levels are indicated by z-scores showing ranges between maximal (green) and minimal values (pink).

In the main analysis, eighteen candidate genes showed similar expression levels in Broca’s and Wernicke’s regions (*p* > 0.05; Table 2). To allow an exploratory classification of these candidates in above-average and below-average expression, we calculated a mean value over all genes using their z-scores from Broca’s and Wernicke’s regions (*x̄* = 0.125) and found twelve genes expressed above-average (overall mean > 0.125): *ATP2C2, CMAHP, CMIP, CNTNAP2, CYFIP1, DCDC2, DNAAF4, FOXP2, NIPA2, RBFOX2, RIPOR2*, and *TUBGCP5*, while *DCDC2* showed the highest expression (overall mean = 0.573; Table 2). Three genes were differentially expressed between both regions: *ATP2C2* showed a higher expression level in Broca’s region as compared to Wernicke’s region (*p* = 0.0291). *DNAAF4* (*p* = 0.0001) and *FOXP2* (*p* = 0.0006) showed higher expression levels in Wernicke’s region, both were stable against correction for multiple comparisons (*p*_FWE_ < 0.05). The fine-mapped expression patterns of the three differentially expressed genes are shown in Figure 4.

**TABLE 2:** Results of the main expression analysis of phonology-related genes.

Gene	Expression levels (mean z-score and standard deviation)			*p*-value
	Broca’s region (*n*_tissue samples_ = 25)	Wernicke’s region (*n*_tissue samples_ = 17)	Overall mean	
*DNAAF4* ^1^	−0.024 ± 0.193	0.353 ± 0.262	0.129 ± 0.290	0.0001*
*FOXP2*	0.270 ± 0.321	0.528 ± 0.254	0.375 ± 0.322	0.0006*
*ATP2C2*	0.501 ± 0.242	0.182 ± 0.337	0.372 ± 0.325	**0.0291**
*ZNF385D*	0.076 ± 0.137	0.012 ± 0.320	0.050 ± 0.231	0.1046
*NOP9* ^2^	−0.237 ± 0.683	−0.082 ± 0.732	−0.174 ± 0.707	0.1533
*MYO18B*	−0.263 ± 0.464	−0.071 ± 0.657	−0.185 ± 0.558	0.1907
*RBFOX2* ^3^	0.272 ± 0.356	0.139 ± 0.509	0.218 ± 0.430	0.2055
*NIPA1*	−0.437 ± 0.494	−0.292 ± 0.459	−0.378 ± 0.478	0.2261
*CMAHP* ^4^	0.469 ± 0.241	0.346 ± 0.319	0.419 ± 0.280	0.2643
*DCDC2*	0.593 ± 0.624	0.543 ± 0.563	0.573 ± 0.601	0.2655
*RIPOR2* ^5^	0.335 ± 0.240	0.433 ± 0.350	0.375 ± 0.293	0.2812
*NIPA2*	0.444 ± 0.405	0.300 ± 0.303	0.386 ± 0.374	0.2856
*CYFIP1*	0.222 ± 0.366	0.402 ± 0.477	0.294 ± 0.424	0.4179
*CMIP* ^6^	0.065 ± 0.479	0.126 ± 0.432	0.090 ± 0.461	0.4207
*ROBO1*	−0.409 ± 0.331	−0.373 ± 0.400	−0.394 ± 0.361	0.4632
*CCDC136*	−0.309 ± 0.394	−0.368 ± 0.574	−0.332 ± 0.476	0.5643
*CNTNAP2*	0.507 ± 0.241	0.411 ± 0.305	0.468 ± 0.273	0.6852
*COL4A2*	0.088 ± 0.563	0.222 ± 0.384	0.142 ± 0.503	0.6864
*FLNC*	−0.281 ± 0.536	−0.298 ± 0.667	−0.288 ± 0.592	0.7665
*TUBGCP5*	0.500 ± 0.328	0.493 ± 0.422	0.497 ± 0.369	0.827
*KIAA0319*	−0.041 ± 0.266	−0.079 ± 0.259	−0.056 ± 0.264	0.8784

*Bold: significant p-value (uncorrected).*

*^∗^*P*-value stable against correction for multiple comparisons using FWE.*

*Previous and alias symbols: ^1^*DYX1C1*; ^2^*C14orf21*; ^3^*RBM9*; ^4^*CMAH*; ^5^*FAM65B*; ^6^*TCMIP* and *KIAA1694*.*

Two types of follow-up analyses were performed. The regional specificity analysis in the frontal lobe found significantly higher expression levels in Broca’s region for *CNTNAP2* (*p* = 0.001) and *DCDC2* (*p* = 0.002), both *p*_FWE_ < 0.05, compared with the language unspecific premotor area (areas 6d1-6d3; Supplementary Table 9A); in the temporal lobe, Wernicke’s region vs. fusiform gyrus (areas Fg1-Fg4) yielded no significant differential expression (*p* > 0.05; Supplementary Table 9B). The control analysis tested random and color genes, each, in Broca’s versus Wernicke’s regions. Neither of the two analyses demonstrated a differential expression stable against correction (both *p*_FWE_ > 0.05; Supplementary Tables 9C,D).

## Discussion

Prior studies have been looking for links between risk genes and phonological aspects in individual disorders. Here we went beyond this point and addressed a more fundamental question, i.e., to identify cross-disease candidate phonological genes whose activity (expression level) we wanted to characterize in key regions of the language network using brain atlas-guided expression mapping. To achieve this, our approach comprised three steps. A systematic literature review that identified 21 phonology-related genes for developmental language and/or learning disorders. An expression analysis found that 12 of these genes were active in Broca’s and Wernicke’s regions, with two genes being more active in Wernicke and one gene being more active in Broca suggesting functional specialization. An enrichment analysis that found out that nine of these genes may contribute to processes of *learning* and *neuronal development.*

### Literature Evaluation Identified Candidate Genes for Three Disorders

Our literature evaluation was designed on the assumption that phonological processing may have a common biological basis in the selected disorders, i.e., the “gene overlap hypothesis.” At the behavioral level, for example, impairments in working memory (Attout and Majerus, 2015; Gray et al., 2019; Johnson et al., 2020) and in learning ability (Rogalski et al., 2008; Bishop, 2009; Landerl et al., 2009) are evident in dyslexia, dyscalculia, SLI, and lvPPA, in addition to phonological impairment. These aspects are closely linked to phonology. However, since mainly linguistic variables of these disorders were investigated in the underlying studies, there is evidence that all genes can be assigned as phonology-related, although it cannot be assumed that this would be exclusively the case.

Because we based our candidate gene search on findings in cohorts of European ancestry, we had to ignore some potentially interesting candidates from the recent literature. One example was a GWAS of rapid automatized naming and rapid alternating stimulus conducted in Hispanic American and African American cohorts (Troung et al., 2019). Both parameters are considered as predictors for reading disability and may be relevant for investigating phonological processing. However, we could not consider the genes *RPL7P34* and *RNLS* from this study. The reason is that genomic and transcriptomic variability (in normal and diseased individuals) may be influenced by population stratification, especially common variation is sensible to this, e.g., SNPs mapping to genes or influencing expression. To avoid this bias, we wanted to keep the data sources (study cohorts) as homogenous as possible.

The gene extractions from the consecutive computational and manual text mining were put forward to the overlap analysis (Figure 3 and Supplementary Table 7C) which revealed that the number of common phonology-related genes was 13 for dyslexia/SLI, six for dyslexia/dyscalculia, and two for dyslexia/dyscalculia/SLI, resulting in 21 genes in total (Supplementary Table 7D). Hence, three disorders contributed to this result, wherein dyslexia was connected to each phonology-related gene. The fourth language disorder, lvPPA, did not share any of its four genes (*APOE, APP, GRN, MAPT*) with the other disorders. LvPPA is a rare disease in the population and only a small number of putative risk genes are known yet (Gorno-Tempini et al., 2004, 2008). This is an important result and does not contradict our initial hypothesis. We consider it rather informative that the genuine developmental disorders with phonological core aspects cluster together and are somewhat distinct from a neurodegenerative disorder with acute phonological but developmentally more general learning deficits (Gorno-Tempini et al., 2008). We have examined lvPPA with the other disorders because there are two arguments from a clinical point of view: First, lvPPA shows clear phonological symptoms, which is why it belongs to the logopedic disorders. Second, Rogalski et al. (2008), for example, showed that patients with PPA are often affected by learning disorders earlier in life before the late-onset of PPA. The chance of a gene overlap with the more polygenic developmental disorders was therefore smaller. Overall, our evaluation supports the view of different molecular processes underlying lvPPA compared to the investigated developmental disorders.

### Gene Enrichment Analysis Implicated Learning and Neuronal Development

Besides the fact that phonological processing is affected in developmental language and/or learning disorders, phonology is the motor for various language learning processes including writing (Treiman, 2017). To explore if phonology-related genes expressed in Broca’s and Wernicke’s regions acting together in the same Biological Processes, Molecular Functions, and Cellular Components, we performed an enrichment analysis using annotations of gene functions (GO terms). The overall results highlight relationships that can be summarized under the topics *learning* and *neuronal development.* Our findings may strengthen the links between phonology and behavioral and/or clinical aspects of the three investigated disorders.

Regarding *learning*, the most significant GO term was “response to auditory stimulus” referring to a process that results in a change in state or activity of a cell or an organism, e.g., gene expression, as a result of an auditory stimulus pointing to the aspect of hearing. In general, auditory processing forms the basis for phonological processing. In case of decreasing auditory performance caused by acquired hearing impairment, studies report a negative influence on phonological representations, for instance, a high working memory capacity was a compensating factor for negative impact of auditory deprivation on phonological processing abilities (Classon et al., 2013). Similar results were found for dyslexia and SLI. In fact, both disorders showed a substantial overlap concerning auditory processing and phonological skills (Fraser et al., 2010). For dyslexia, a link between hearing and reading abilities is known, i.e., changes in brain networks involved in phonological processing lead to deficits in matching speech sounds to their corresponding visual representations (Wallace, 2009).

Another finding of the topic *learning* provides a link between the term “vocal learning,” the transcription factor gene *FOXP2*, and one of its prominent transcriptional targets, the cell adhesion and receptor gene *CNTNAP2*, both contributing to all GO terms of the topic *learning*. Since the genes occur in five of eight outcomes, it can be concluded that both genes exert some dominance on result generation. A reason could be that there is plenty of experimental data on both in the literature and databases compared to other genes of our investigation. As *FOXP2* is important in human language development and its disorders, its avian homolog *FoxP2* is thought to play a crucial role in vocal learning and possible deficits in song-learning birds (Jarvis, 2019). C*NTNAP2* was associated with neurodevelopmental disorders including language deficits (Vernes et al., 2008) and variation in language development in the general population (Whitehouse et al., 2011). Recent results suggested that *CNTNAP2* is involved in a developmental cascade of effects in infants where it controls rapid auditory processing leading to early expressive language (Riva et al., 2018).

When comparing the two above-mentioned terms “response to auditory stimulus” and “vocal learning,” one gene (*KIAA0319*) only occurred in the term “response to auditory stimulus” and hence was probably the gene that distinguishes both findings. This observation may be interesting since a knock down of *Kiaa0319* expression in a rat model altered cortical responses and sound processing in the auditory cortex which was taken as an analogy for phoneme processing in humans (Centanni et al., 2014). *KIAA0319* encodes a cell adhesion molecule involved in development of the cerebral cortex. In fact, the gene mediates adhesion between migrating neurons and radial glial fibers as well as it regulates the morphology of dendrites (Peschansky et al., 2010). On the question of *KIAA0319* being functionally related to *FOXP2*, there is little literature. In a fMRI study by Pinel et al. (2012) in a reading task in healthy subjects, they found evidence that common genetic variations (SNPs) in both genes may play an important role in language development and in normally developed language networks, but in different cerebral pathways: two *FOXP2* SNPs were associated with variations of activation in the left frontal cortex, while one *KIAA0319* SNP was associated with asymmetry in functional activation of the superior temporal sulcus.

The GO term “dendrite development” was the second most significant enrichment result and addresses the progression of dendrites, from its formation to a mature structure. Five of our candidate genes contributed to this term (*CYFIP1*, *DCDC2*, *KIAA0319*, *RBFOX2*, *ROBO1*). Dendritic extensions propagate electrochemical signals received from other neural cells to the cell body of the neuron. These issues are important constituents of learning and behavior, including synaptic plasticity, and therefore build a strong content-related connection to our first topic (*learning*). *CYFIP1* coordinates the translation of messenger RNA at dendrites (Crespi, 2017). High expression of *CYFIP1* due to gene duplication has been associated with autism (Oguro-Ando et al., 2015). Conversely, low expression by gene deletion was associated with risk of schizophrenia and impaired cognition among otherwise-neurotypical individuals (Stefansson et al., 2008, 2014; Tam et al., 2010). The deletion also affects cognitive, structural, and functional correlates of dyslexia as well as dyscalculia and mediates the strongest risk to the combined disorders (Ulfarsson et al., 2017). A GWAS found association between reading and language skills and SNP rs5995177 in *RBFOX2* (Gialluisi et al., 2014). It encodes an alternative splicing regulator (RNA binding fox-1 homolog 2) involved in brain development (Gehman et al., 2012). A follow-up candidate gene study found a generalized effect of the SNP on cortical thickness in reading- and language-related brain regions of healthy adults (Gialluisi et al., 2017). The rodent homolog of *ROBO1* (*Robo1*) encodes the roundabout guidance receptor 1 which is known for regulating the midline crossing of commissural nerve tracts, a process essential for mammalian brain formation. In family pedigrees from Finland, a rare *ROBO1* haplotype was identified which dominantly co-segregates with the dyslexia diagnosis (Hannula-Jouppi et al., 2005). Individuals who carried this *ROBO1* variant showed dose-dependent impaired interaural interaction suggesting that crossing of the auditory pathways requires an adequate *ROBO1* expression during development (Lamminmäki et al., 2012). *DCDC2* and *KIAA0319* provide experimental evidence for a dual role in both topics. In *neuronal development*, *DCDC2* (doublecortin domain-containing protein 2) is involved in regulating neuronal migration, especially for the cerebral cortex (Galaburda et al., 2006). In *learning*, *DCDC2* was associated with phonological awareness and auditory phonological representations (Goswami, 2011; Eicher et al., 2015). *KIAA0319* (neuronal migration) mediates axon guidance as well as dendrite formation. In this respect, *DCDC2* and *KIAA0319* are among the most studied genes in language and/or learning disorders and future studies of phonological processing that aggregate variation of all five genes seem to be worthwhile.

### Expression Analysis Revealed Putative Roles of *ATP2C2*, *DNAAF4*, and *FOXP2*

Expression differences of a gene in two language network regions are not direct evidence for a functional involvement of this gene in phonological processing. However, this arises from combining expression mapping and gene function associated with phonology determined in previous studies. The same is true for associations with disorders, because the expression data from the Allen Human Brain Atlas and mapping data from the Julich-Brain Atlas were collected in brains of people who had no history for neurological or psychiatric disease. Since the Allen expression data do not provide additional genotype data, we cannot directly examine the relationship between genome (genotype), transcriptome (RNA), and phenotype (disease or imaging trait like gray matter volume).

Regarding the main analysis, we found evidence for a significant differential expression of three phonology-related genes. In fact, expression of *ATP2C2* was upregulated in Broca’s region, while expression of *DNAAF4* and *FOXP2* was upregulated in Wernicke’s region. The regional specification analyses for the frontal and the temporal lobes showed that almost all phonology-related genes, including our top findings, were not differentially expressed between Broca’s region and premotor region (6d1–6d3) in the frontal lobe, and between Wernicke’s region and fusiform gyrus (Fg1-Fg4) in the temporal lobe (Supplementary Tables 9A,B). The only follow-up analysis showing a significant and FWE correction-stable expression difference, was the frontal lobe comparison, revealing a higher expression of *CNTNAP2* and *DCDC2* in Broca suggesting their activity in this part of the language network. Because none of our candidate genes was significantly upregulated after correction in the non-language areas, we hypothesize that *ATP2C2, DNAAF4*, and *FOXP2* were specifically expressed in cortical regions being language and/or phonology relevant. To further evaluate the biological specificity of these results, we performed two additional control analyses. Neither the genes of a random selection from the whole transcriptome nor the genes of human body coloration achieved a correction stable result. Both negative results, together with knowledge about the disorders, brain regions, and gene functions, supported the specificity of regional expressions of *ATP2C2, DNAAF4*, and *FOXP2*.

The literature analysis suggested an overlap between dyslexia and SLI for *ATP2C2* (Newbury et al., 2011; Müller et al., 2017; Martinelli et al., 2021). In the enrichment analysis, it did not show up among our top findings but provided nominal significance in a gene set with *ROBO1* for a non-brain related biological process (Supplementary Table 8). Different studies provided evidence that *ATP2C2* is involved in phonological processing, for instance, regarding a modulation of phonological working memory in language impairment (Newbury et al., 2009; Smith et al., 2015). *ATP2C2* encodes a magnesium-dependent calcium transporter. Regarding a translation of our *ATP2C2* finding to a clinical level, it seems to be important that imbalances of magnesium and calcium ions in blood serum were reported in patients with dyslexia and other communication disorders (Kurup and Kurup, 2003).

For *DNAAF4*, the literature analysis provided a link between dyscalculia and dyslexia. In the enrichment analysis, the gene was part of the GO term “cognition” highlighting the topic *learning*. *DNAAF4*, formerly known as *DYX1C1* (dyslexia susceptibility 1 candidate 1), was described as axonemal dynein assembly factor required for ciliary motility (Tarkar et al., 2013). More relevant for our study was a previous finding of a protein-protein interaction with estrogen receptors possibly linking its well-known role in neuronal migration during cortical development to an involvement of hormonal pathways in the development of dyslexia (Wang et al., 2006; Massinen et al., 2009). In this line, *DNAAF4* was found to be associated with phonological memory in dyslexia (Lim et al., 2014) as well as with phonological working memory in healthy participants (Marino et al., 2007). Of note, knockdown of *Dnaaf4* in rat embryos demonstrated effects on auditory processing, visual attention as well as cortical and thalamic anatomy (Szalkowski et al., 2013). In humans, two SNPs, rs17819126 in *DNAAF4* and rs8053211 in *ATP2C2*, represented the only correction stable results in an association screen of ten candidate genes tagged by 23 independent SNPs for event-related potential mismatch response, an indicator for auditory discrimination capabilities in dyslexia patients (Müller et al., 2017). Müller et al. (2017) provided evidence that genetic variation in *ATP2C2* and *DNAAF4* may be a putative modulator of mismatch response in dyslexia. This aspect is highly interesting since auditory discrimination is an essential prerequisite for phonological processing and should be investigated in a future study, especially in context with disease-dependent changes of structural and functional connectivity between Broca’s and Wernicke’s regions.

*FOXP2* is considered to be one of the most important genes for speech development and disorder. Its relevance was hypothesized when rare variation (a missense mutation) was discovered in members of a British family with frequent problems in articulating, formulating, and understanding of speech (Lai et al., 2001). Subsequently, common variation (SNPs) was investigated at this gene locus and associations between SNPs and dyslexia as well as other language and/or learning disorders were identified (Mascheretti et al., 2017). *FOXP2* expression is relevant during brain development, when the transcription factor controls the transcriptional activity of different target genes, e.g., *CNTNAP2* (Castro Martínez et al., 2019). Our literature evaluation provided a link between *FOXP2* and dyslexia and SLI (Eicher et al., 2013; Doust et al., 2020). In our enrichment analysis, the gene contributed to the best findings of the topic *learning*. The significantly higher expression of *FOXP2* in Wernicke’s region may fit to a finding of a study by Wilcke et al. (2012). The authors report that the dyslexia-associated SNP rs12533005 in *FOXP2* was associated with grapheme-phoneme correspondence abilities (linking letters to speech sounds) in written language in an inferior parietal area near Wernicke’s region involved in phonological processing (Wilcke et al., 2012). We speculate whether the higher activity of *FOXP2* in Wernicke’s region could support the concept of regional specificity. Currently, *FOXP2* is mainly known as a gene linked to childhood apraxia of speech and speech motor planning deficits (Morgan et al., 2016). The possible link between *FOXP2* and phonology could be a new aspect to investigate further in the future.

### Limitations

The search criteria (“clinical categories”) of the literature evaluation were rather broad to cover the terminological variability between published studies of the four different disorders as much as possible. Consequently, we had to make the compromise of obtaining results at the genomic level that contained broader findings in addition to the desired disorders, e.g., genes for arithmetical weakness in case of dyscalculia. Basically, we only extracted markers and genes from a study that were self-reported as best findings by the study authors. We specified *p* < 0.05 for criterion (c) as a general threshold for significance; for GWAS, we tried to stick to genome-wide significant findings (*p* < 5 × 10^–8^) if reported. Thus, we always stayed within the logic of the respective study and did not make an arbitrary decision for or against a selection. This is the reason why we did not consider negative findings in our study. Many phonology-related genes have been selected from traditional candidate gene studies rather than large studies such as GWAS. The main reason is that we studied four disorders for which the literature is very diverse. Some disorder phenotypes already have GWAS, while others do not yet. Therefore, we did not prioritize study types in the design (GWAS, candidate gene study, linkage study). This may be a limitation, but in practice it was not feasible otherwise. Therefore, our gene selection may be interpreted with caution. To minimize the risk of unrecognized studies and genes, we performed a supporting search in the GWAS Catalog database for common genetic variants and their mapped genes across the four disorders during the period of the primary literature search (2010–2021). Despite thorough mining, we could not prevent the selection of gene markers with weak replication evidence in independent studies, e.g., *RBFOX2*. Again, we urge caution in interpreting the results. Ultimately, only meta-analyses will help to separate the robust associations from the false positives.

The assumption of the enrichment analysis was that the phonology-related genes we have yet to find will be consistent with what is already known about phonological processing and/or its genetic basis which may not always be true. The enrichment analysis can only be as accurate as the underlying annotations (GO categories) since not all genes, especially in humans, have phenotype annotations and there are many genes whose functions are not defined yet. As a result of these limitations, enrichments may arise in GO terms where certain genes show noticeable dominance that may bias interpretation, e.g., *CNTNAP2* and *FOXP2* in the terms of the topic “learning.” This is a caveat to keep in mind when interpreting results.

In the expression analysis, we focused on Broca’s and Wernicke’s regions. This does not mean that we ignore the broader network including the subcortical regions from which we did not include basal ganglia and striatum because these structures are associated with speech motor processes rather than phonology (Eickhoff et al., 2009). A similar reason applies for the cerebellum which is mainly responsible for speech motor control (Houde and Nagarajan, 2011). Because of the left hemispheric dominance for language for left- and right-handed people (Branch et al., 1964; Mazoyer et al., 2014), and the fact that there is more data for the left hemisphere in the Allen Human Brain Atlas expression data, only data from the left hemisphere were analyzed. Although the exclusive use of left hemispheric data is based on a strong biological assumption, this should still be considered as a possible limitation and taken into account when interpreting the data. Areas 44 and 45 were combined into one single VOI, considering the spatial constraints of availability of gene expression data, which did not allow searching in a more detailed way. At the same time, differences between both areas at the level of cyto- and receptorarchitecture (Amunts et al., 2010) as well as connectivity and function (Anwander et al., 2007; Friederici and Gierhan, 2013) have been described in the past, and the functional specialization of both areas undergoes changes during ontogeny until it is finally differentiated (Amunts et al., 2003; Skeide and Friederici, 2016). Therefore, it can be hypothesized that both areas may also differentially contribute to the findings of the present study. In addition, it cannot be excluded that the detected expression differences between Broca’s and Wernicke’s regions were influenced, to a certain degree, by differences in their normal cell type proportions of the bulk tissue used by Allen Brain. Further studies will be necessary to more precisely elucidate the role of the different cortical areas underlying Broca’s and Wernicke’s regions. The strength of the dataset used is the dense spatial coverage of the tissue samples whose gene expressions were analyzed. Together with the probability-based location information contained in the Julich-Brain maps, a meaningful differential gene expression analysis can be calculated. However, the relatively small number of six donors is also a statistical limitation that would suggest further molecular genetic analyses.

### Conclusion

In the present study, we gathered previously published evidence for genetic factors underlying phonological processing symptoms of the language and learning disorders dyslexia, dyscalculia, and SLI and performed expression fine-mapping in two key regions of the language network. While most previous studies sought to link candidate genes and phonological aspects of a single disorder, we focused on a more general aim, to understand whether a symptom, i.e., phonological processing, may have a common genetic regulation in different disorders. Overall, the identified regional expression of *ATP2C2*, *DNAAF4*, and *FOXP2* together with markers from the literature provide evidence of a putative role not only in impaired phonological processing but also for the healthy subjects. In this regard, our study may be a use-case for larger studies on the genetics of phonological processing in the future. To investigate new hypotheses for such gene-phenotype correlates *in vivo*, one option would be to test for association between DNA variation (SNPs, copy-number variants) at candidate gene loci and effects on phonological processing in cortical areas like Broca’s and Wernicke’s regions, and also in subcortical areas of the language network using large samples of the three disorders and beyond at different ages.

## Data Availability Statement

Data from Allen Human Brain Atlas are available at http://human.brain-map.org/ and data from Julich-Brain are available at https://www.jubrain.fz-juelich.de. A stand-alone version of JuGEx is available at http://www.fz-juelich.de/inm/inm-1/jugex. JuGEx is also part of the Atlas of the Human Brain Project, available at https://www.humanbrainproject.eu/en/explore-the-brain/atlases/.

## Ethics Statement

The study computationally analyzed data from the Allen Human Brain Atlas and Julich-Brain Atlas. For Allen Brain: Brain tissues were collected after obtaining informed consent from the decedent’s next-of-kin. Institutional Review Board review and approval were obtained for the collection of tissue and non-identifying case information at the tissue banks and repositories that provided tissue for this project (http://help.brain-map.org/display/humanbrain/Documentation). For Julich-Brain: The brains were obtained through the body donor program of the Department of Anatomy at the University of Düsseldorf in accordance with the rules of the local ethics committee (#4863). The patients/participants’ next of kind provided their written informed consent to participate in this study.

## Author Contributions

NU, DIH, and TWM conducted analyses. NU, SH, KA, and TWM analyzed and interpreted the results. SB supported the JuGEx analyses. PP provided digital versions of the cytoarchitectonic probability maps. SH, SC, KA, and TWM supervised all aspects of the study. NU wrote a first draft of the manuscript. All authors critically revised the manuscript and approved its publication.

## Conflict of Interest

The authors declare that the research was conducted in the absence of any commercial or financial relationships that could be construed as a potential conflict of interest.

## Publisher’s Note

All claims expressed in this article are solely those of the authors and do not necessarily represent those of their affiliated organizations, or those of the publisher, the editors and the reviewers. Any product that may be evaluated in this article, or claim that may be made by its manufacturer, is not guaranteed or endorsed by the publisher.

## Footnotes

1
https://pubmed.ncbi.nlm.nih.gov/advanced

2
https://www.ncbi.nlm.nih.gov/research/pubtator

3
https://www.genenames.org

4
http://bioinformatics.psb.ugent.be/webtools/Venn/

5
https://toppgene.cchmc.org/enrichment.jsp

6
https://www.ebi.ac.uk/QuickGO/

7
https://www.fz-juelich.de/inm/inm-1/jugex

8
https://search.kg.ebrains.eu/

## Supplementary Material

Supplementary Table 1 Overview of phonological deficits in the four investigated disorders.

Supplementary Table 2 Overview of studies on Broca’s and Wernicke’s regions in humans and model organisms.

Supplementary Table 3 Literature evaluation—The eleven single searches using PubMed Advanced Search.

Supplementary Table 4 Literature evaluation—PubMed Advanced Search combining clinical categories and genetic terms for the identification of phonology-related genes.

Supplementary Table 5 Literature evaluation—Clinical categories found in research articles.

Supplementary Table 6A Gene expression analysis—Random genes used as negative control.

Supplementary Table 6B Gene expression analysis—Color genes used as negative control.

Supplementary Table 7A Literature evaluation—The 36 excluded review articles.

Supplementary Table 7B Literature evaluation—The 71 research articles providing the 77 candidate genes.

Supplementary Table 7C Literature evaluation—The 77 candidate genes from the remaining 71 research articles.

Supplementary Table 7D Literature evaluation—Basic annotation of the 21 phonology-related genes.

Supplementary Table 7E Literature evaluation—The genetic items extracted from the 21 phonology-related genes.

Supplementary Table 8 Total results of the functional enrichment analysis of phonology-related genes.

Supplementary Table 9A Follow-up gene expression analysis in the frontal lobe—Phonology-related genes—Broca’s region vs. premotor areas.

Supplementary Table 9B Follow-up gene expression analysis in the temporal lobe—Phonology-related genes—Wernicke’s region vs. fusiform gyrus.

Supplementary Table 9C Follow-up gene expression analysis—Random genes—Broca’s region vs. Wernicke’s region.

Supplementary Table 9D Follow-up gene expression analysis—Color genes—Broca’s region vs. Wernicke’s region.
